# Immunotherapy utilizing the combination of natural killer– and antibody dependent cellular cytotoxicity (ADCC)–mediating agents with poly (ADP-ribose) polymerase (PARP) inhibition

**DOI:** 10.1186/s40425-018-0445-4

**Published:** 2018-11-29

**Authors:** Kathleen E. Fenerty, Michelle Padget, Benjamin Wolfson, Sofia R. Gameiro, Zhen Su, John H. Lee, Shahrooz Rabizadeh, Patrick Soon-Shiong, James W. Hodge

**Affiliations:** 1 Laboratory of Tumor Immunology and Biology, Center for Cancer Research, National Cancer Institute, National Institutes of Health, 10 Center Drive, Room 8B09, Bethesda, MD 20892 USA; 2 EMD Serono, Billerica, MA USA; 3 NantOmics, Culver City, CA USA

**Keywords:** PARP inhibitors, ADCC, Prostate carcinoma, BRCA, TRAIL

## Abstract

**Background:**

Poly (ADP-ribose) polymerase inhibitors (PARPi) prevent single-stranded DNA repair. Olaparib is a PARPi approved for the treatment of BRCA mutant ovarian and breast carcinoma. Emerging clinical data suggest a benefit of combining olaparib with immunotherapy in prostate cancer patients both with and without somatic BRCA mutations.

**Methods:**

We examined if olaparib, when combined with IgG_1_ antibody-dependent cellular cytotoxicity (ADCC)-mediating monoclonal antibodies (mAbs) cetuximab (anti-EGFR), or avelumab (anti-PD-L1), would increase tumor cell sensitivity to killing by natural killer (NK) cells independently of BRCA status or mAb target upregulation. BRCA mutant and BRCA wildtype (WT) prostate carcinoma cell lines were pretreated with olaparib and then exposed to NK cells in the presence or absence of cetuximab or avelumab.

**Results:**

NK-mediated killing was significantly increased in both cell lines and was further increased using the ADCC-mediating mAbs. Pre-exposure of NK cells to recombinant IL-15/IL-15Rα further increased the lysis of olaparib treated tumor cells. In addition, olaparib treated tumor cells were killed to a significantly greater degree by engineered high-affinity NK cells (haNK). We show here for the first time that (a) olaparib significantly increased tumor cell sensitivity to NK killing and ADCC in both BRCA WT and BRCA mutant prostate carcinoma cells, independent of PD-L1 or EGFR modulation; (b) mechanistically, treatment with olaparib upregulated death receptor TRAIL-R2; and (c) olaparib significantly enhanced NK killing of additional tumor types, including breast, non-small cell lung carcinoma, and chordoma.

**Conclusions:**

These studies support the combined use of NK- and ADCC-mediating agents with correctly timed PARP inhibition.

**Electronic supplementary material:**

The online version of this article (10.1186/s40425-018-0445-4) contains supplementary material, which is available to authorized users.

## Background

Poly (ADP-ribose) polymerase inhibitors (PARPi) are FDA approved for use in ovarian and breast carcinoma with the mutant breast cancer susceptibility gene (*BRCA*). Data suggest that the mechanism of action in these tumors is synthetic lethality, wherein the PARP inhibitors block the recruitment of the requisite base excision DNA repair pathway machinery and defects in BRCA simultaneously prevent homologous recombination, ultimately leading to genomic instability. The accumulation of DNA damage triggers cell cycle arrest, DNA repair, and potentially apoptosis [1]. Gasser et al., reported that genotoxic stress and stalled DNA replication forks induce the expression of ligands for the NKG2D receptor found in natural killer cells [1], suggesting that drugs inhibiting DNA repair may augment NK killing.

Recent clinical data has sparked interest in the immunomodulatory potential of PARP inhibitors regardless of BRCA status and, in particular, in combination therapies with checkpoint inhibitors. An ongoing phase 2 study of olaparib and the programmed death-ligand 1 (PD-L1) inhibitor durvalumab in metastatic castration-resistant prostate cancer (mCRPC) patients demonstrated that 8/17 patients (47%) had a decrease in prostate-specific antigen (PSA) of > 50%, with two of those patients having no known mutations in DNA damage response pathways [2]. It should be noted that the Fc region of durvalumab has been modified in such a way that it does not induce either antibody-dependent cytotoxicity (ADCC) or complement-dependent cytotoxicity (CDC).

We interrogated the immunomodulatory potential of the PARPi olaparib in vitro*,* focusing on prostate carcinoma. We hypothesized that olaparib would increase target cell sensitivity to killing by human natural killer (NK) cells independent of BRCA status or ADCC mAb target modulation. We used two prostate carcinoma cell lines: 22RV1, which has known deleterious BRCA2 mutations, [3] and DU145, which does not have known deleterious mutations in either BRCA1 or BRCA2 [4]. BRCA status of these lines was independently confirmed using next generation sequencing (Dr. Paul Meltzer, M.D., Ph.D., NCI, NIH).

Combination therapies utilizing PARPi also have implications beyond the use of patients’ native immune system. High-affinity NK (haNK) cells are an NK cell line, NK-92, which has been engineered to endogenously express IL-2 as well as the high-affinity valine (V) CD16 allele [5]. Here, we use haNK in combination with PARPi and antibody-dependent cellular cytotoxicity (ADCC)-mediating antibodies to increase target cell lysis.

Our data show for the first time that (a) olaparib significantly increased tumor cell sensitivity to NK-mediated killing and ADCC in both BRCA WT and BRCA mutant prostate carcinoma cells, independent of PD-L1 or epithelial growth factor receptor (EGFR) modulation; (b) olaparib treatment significantly enhanced NK killing in a variety of tumor types, including prostate, breast, and non-small cell lung carcinoma as well as chordoma; and (c) mechanistically, treatment with olaparib upregulated death receptor TRAIL-R2. These studies support the combined use of NK- and ADCC-mediating agents with PARPi in BRCA mutant and WT prostate carcinoma as well as other tumor types.

## Methods

### Tumor cell lines

Human prostate tumor cell lines (22RV1 and DU145), breast cancer (MCF7) and lung cancer (H460) were obtained from American Type Culture Collection (Manassas, VA). Triple negative breast carcinoma (SUM149) was obtained from Asterand Biosciences (Detroit, MI). Chordoma cells (Ch22) were generously supplied by The Chordoma Foundation (Durham, NC). DU145 TNFRSF10B (TRAIL Receptor 2) CRISPR knockout and corresponding wild type cell pools were obtained from Synthego (Menlo Park, CA). Removal of TRAIL R2 in DU145 TNFRSF10B −/− cells was validated by Synthego via genome sequencing against wild type cells and confirmed by flow cytometry. All cell lines were passaged for less than 6 months, free of *Mycoplasma* and cultured at 37 °C/5% CO_2_. 22RV1 and H460 were maintained in RPMI, DU145 were maintained in EMEM, Ch22 were maintained in DMEM, MCF7 were maintained in DMEM supplemented with insulin (2.5 μg/mL), and SUM149 were maintained in Ham’s F12 supplemented with insulin (2.5 μg/mL) and hydrocortisone (1 μg/mL). All media were supplemented with 10% fetal bovine serum, 1% penicillin/streptomycin, 0.5% gentamicin, nonessential amino acids (final concentrations: L-alanine (8.9 mg/L), L-asparagine (15 mg/L), L-aspartic acid (13.3 mg/L), L-glutamic acid (14.7 mg/L), glycine (7.5 mg/L), L-proline (11.5 mg/L), L-serine (10.5 mg/L)), and L-glutamine (final concentration 2 mM).

### Human healthy-donor NK cells

Blood samples were obtained from normal healthy donors on the NCI IRB approved NIH protocol 99-CC-0168. Research blood donors were provided written informed consent. Donors were non-pregnant adults of 18 years or older, who met healthy blood donor criteria and tested negative for transfusion-transmissible diseases. Blood was collected by standard phlebotomy and apheresis techniques in ACD-A (Anticoagulant Citrate Dextrose Solution Formula A), and the cells were mixed with the donor’s plasma. All samples were de-identified. PBMCs were isolated from this apheresis product within 24 h of donor sample collection using gradient centrifugation with Lymphocyte Separation Medium (Mediatech, Manassas, VA). Cells were washed with PBS (Mediatech) and adjusted to a concentration of 5 × 10^7^ cells/ml with Fetal Bovine Serum (Atlanta Biologicals, Atlanta, GA) containing 10% DMSO prior to freezing. Median cell yield of PBMCs prior to freezing was typically 1 × 10^9^ total cells with 90–95% viability as determined by trypan blue exclusion. Cells were cryopreserved using CoolCell LX (Corning, Corning, NY) freezing containers at a cooling rate of 1 degree Celsius per minute in a − 80 degree Celsius freezer for 24 h, then placed in Liquid Nitrogen (vapor phase) for long-term storage. Median cell yield after thawing one 5 ml vial (frozen at 5 × 10^7^ cells/ml) was typically 1.5–2 × 10^8, with 90–98% viability. NK effector cells were isolated from PBMCs using the Human NK Cell Isolation (negative selection) Kit 130–092-657 (Miltenyi Biotech, San Diego, CA), according to the manufacturer’s protocol. Median NK cell yield after isolation was typically 0.5-1 × 10^7^ with 94–98% viability. Purified NK cells were incubated overnight in RPMI-1640 medium (Mediatech, Manassas, VA) containing 10% fetal bovine serum (Gemini Bio-Products, West Sacramento, CA), glutamine, and antibiotics (Mediatech) prior to use. Fetal bovine serum was pretested for support of NK proliferation and function. Median NK cell yield after overnight resting was typically 5-8 × 10^6^ with 90–96% viability.

### High-affinity NK (haNK) cells

High-affinity NK (haNK) cells are an NK cell line, NK-92, which has been engineered to express IL-2 and the high-affinity valine (V) CD16 allele as previously described [5–7]. haNK cells were supplied by NantBioScience (Culver City, CA) through a Cooperative Research and Development Agreement (CRADA) with the National Cancer Institute (NCI) and cultured in X-Vivo-10 medium (Lonza, Walkersville, MD) supplemented with 5% heat-inactivated human AB serum (Omega Scientific, Tarzana, CA) at a concentration of 5 × 10^5^ cells/ml.

### Antibodies

Avelumab and matching IgG1 isotype control were obtained from EMD Serono (Rockland, MA) as part of a CRADA with the NCI as previously described [8, 9], and used at 2 μg/ml final concentration. Matching IgG1 cetuximab was obtained from Bristol Myers-Squibb (New York, NY), and was used at 1 μg/ml final concentration. Anti-human CD16-neutralizing mAb was obtained from Thermo Fisher Scientific (Waltham, MA). Anti-TRAIL-R antibody (KillerTRAIL) was obtained from Enzo Life Sciences (Farmingdale, NY). For Western blot, cell lysates were stained with antibodies for phospho-TBK1 (Cell Signaling, Danvers, MA #4947 s), TBK1 (Cell signaling #3504), IRF-3 (Cell Signaling #3013), phospho-IRF3 (Cell Signaling #5483 s), cGAS (Sigma-Aldrich, HPA031700), phospho-STING (Cell Signaling #85735) and vinculin (Sigma Aldrich, v9131). Rabbit polyclonal antibody targeting residues 324–340 of human STING was a kind gift from Glen N. Barber, Ph.D., University of Miami, Miami, FL. Secondary antibodies used were IRDye 800CW goat anti-mouse (LI-COR Biosciences, Lincoln, NE) and IRDye 680RD goat anti-rabbit (LI-COR Biosciences).

### Chemicals and drug exposure

Olaparib was obtained from Selleck Biochemical (Houston, TX). For cytotoxic assays, adherent tumor cells in log-growth phase based on real-time cell analysis were exposed for 24 h to olaparib (20 μM). For flow cytometry, Western blots, and NanoString, cells at 50–80% confluency were exposed to olaparib (20 μM) while incubating at 37 °C/5% CO_2_. For select experiments, NK cells were treated with 50 ng/mL IL-15/IL-15Rα superagonist N-803 (formerly known as ALT-803) obtained from Altor BioScience (Miramar, FL, now NantBioScience) as part of a CRADA with the NCI. After drug exposure as described above, cells were harvested, and viable cells were counted by trypan blue exclusion using a Cellometer Auto T4 automated cell counter (Nexcelom Bioscience, Lawrence, MA).

### NK lysis in vitro assay

Prostate carcinoma cell lines (22RV1 or DU145) received 24 h pretreatment with 20 μM olaparib. NK cells were plated at a 5:1 NK to target cell ratio, with or without ADCC-mediating antibodies avelumab (anti-PD-L1) and cetuximab (anti-EGFR). Cell lysis was monitored for 36 h on an xCelligence RTCA impedance-based assay. DU145 TNFRSF10B −/− and wild type cell pools were treated, plated with NK, and monitored using the method described above. In select experiments, NK cells were incubated for 2 h with anti-CD16 antibody (Thermo Fisher Scientific) or Concanamycin A (Sigma-Aldrich, St. Louis, MO).

### Flow cytometry

Tumor cells were cultured as described and treated in the presence or absence of olaparib (20 μM) for 60 h (to replicate the conditions in the assays, 24 h pretreatment and 36 h data collection period). Cells were then harvested and stained with the following antibodies: TRAIL-R2-APC (BD Biosciences, Franklin Lakes, NJ), PD-L1-PE (BD Biosciences), EGFR-PE (BD Biosciences). TRAIL R2 knockout in DU145 TNFRSF10B −/− versus wild type cell pools was confirmed using the same treatment method and stained with TRAIL-R2-PE (BD Biosciences).

NK cells were isolated from PBMCs as described and treated in the presence or absence of olaparib (20 μM) overnight in RPMI-1640 supplemented with 10% FBS. Cells were then stained with the following antibodies: CD16-PE (BD Biosciences), CD56-PE (BD Biosciences), DNAM1-PE (BD Biosciences), NKG2D-PE (BD Biosciences), NKp44-PE (BD Biosciences), NKp46-PE (BD Biosciences), Granzyme-PE (BD Biosciences), Perforin-PE (BD Biosciences).

Cell viability of both tumor and NK cells was examined using trypan blue staining prior to data acquisition on a FACSCalibur flow cytometer. Live cells were gated via forward and side scatter. Data were analyzed using FlowJo software (TreeStar Inc., Ashland, OR). Isotype control staining was < 5% for all samples analyzed. In selected experiments, target cells (22RV1 and DU145) were cultured as described and treated for 24 h with 20 μM olaparib. The tumor cells were then co-cultured at a 1:1 ratio with either NK cells or haNK cells for 24 h prior to staining using the Image-iT LIVE Green Poly Caspases Detection Kit for microscopy (Thermo Fisher Scientific). Per the manufacturer’s protocol, cells were additionally stained for DAPI (Perkin Elmer, Waltham, MA) and CD56-APC (BD Biosciences), which allowed for the exclusion of human NK cells and haNK cells from analysis. Data were acquired on the Amnis Imagestream (EMD Millipore, Burlington, MA).

### Apoptotic array

Cells were treated with 20 μM olaparib for 12 h at 37 °C/5% CO_2_. The Apoptosis RT^2^ Profiler PCR Array (Qiagen, Germantown, MD) was used to identify modulation of genes related to apoptosis according to the manufacturer’s instructions. The raw data tables for the apoptotic array are depicted in Additional file 1: Table S2.

### NanoString array

NanoString data are available in the GEO database under accession number GSE121682. The nCounter® PanCancer Pathways Panel (NanoString Technologies, Seattle, WA) was performed according to the manufacturer’s instructions on 22RV1 and DU145 cells after 6 h, 12 h, and 18 h of treatment with 20 μM olaparib. Seven hundred seventy genes were surveyed as part of this multi-gene, RNA-based analysis and a 3-fold change cutoff was used to identify initial genes of interest. Curated genes were chosen based on both NanoString and apoptotic array data for inclusion in a STRING analysis of protein-protein interactions [10, 11].

### Statistical analysis

Significant differences in the distribution of cell populations by flow cytometry analyses were examined using the Kolmogorov-Smirnov test and considered biologically significant if they differed by ≥20% relative to respective controls or if mean fluorescence intensity (MFI) underwent a 3-fold increase. Significant differences between two treatment groups were determined by a 2-tailed *t* test using GraphPad Prism 7.0 software. Differences were considered significant when the *p* value was < 0.05.

## Results

### NK-mediated lysis of prostate carcinoma cells is increased following exposure to olaparib

**Fig. 1:**
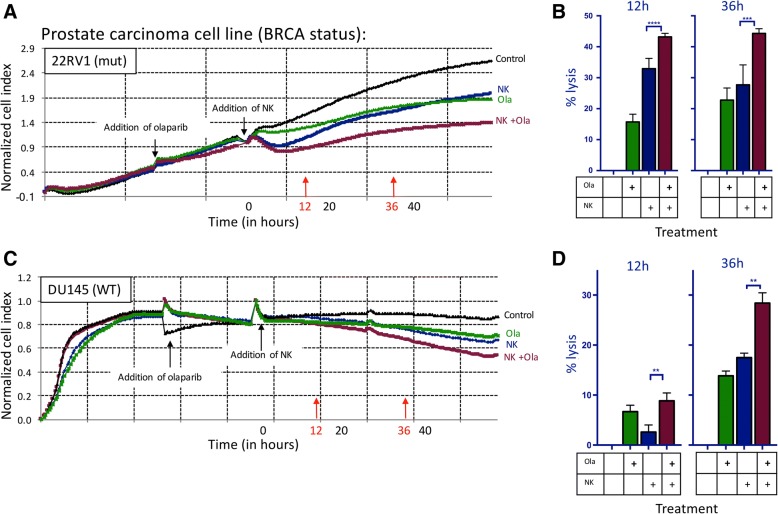
NK-mediated lysis of prostate carcinoma cells is increased following exposure to olaparib. **a** Real-time impedance-based cell analysis of BRCA mutant prostate carcinoma (22RV1) cells in the presence or absence of olaparib (ola) and NK. **b** Lysis of BRCA mutant cells treated with or without olaparib and NK compared to control at 12 h and 36 h. **c** Real-time impedance-based cell analysis of BRCA WT prostate carcinoma (DU145) cells in the presence or absence of olaparib and NK. **d** Lysis of BRCA WT cells treated with olaparib and NK compared to control at 12 h and 36 h. These experiments were performed with four different human NK donors with similar results. *p* < 0.01**, *p* < 0.001***, *p* < 0.0001****

Olaparib is currently FDA approved only for ovarian and HER2-negative breast cancer in patients with germline BRCA mutations [12]. We focused on two prostate carcinoma cell lines, BRCA mutant 22RV1 and BRCA WT DU145; both were exposed to olaparib in vitro. Olaparib exposure significantly increased NK-mediated lysis as assessed by real-time cell analysis (RTCA) (Fig. 1a). NK-mediated killing of treated cells was increased by 1.3-fold compared to olaparib treatment alone in the BRCA mutant cell line 22RV1 after 12 h (*p < 0.0001*) and 1.6-fold difference persisted at 36 h (*p = 0.0001*) (Fig. 1b). Surprisingly, olaparib exposure also significantly increased NK-mediated lysis of the BRCA WT line DU145 (Fig. 1c). Lysis was increased by 3.4-fold (*p = 0.0071*) at 12 h and by 1.6-fold at 36 h (*p = 0.0011*) (Fig. 1d).

### NK-mediated lysis of prostate carcinoma cells treated with olaparib is further increased by addition of ADCC-mediating antibodies

**Fig. 2:**
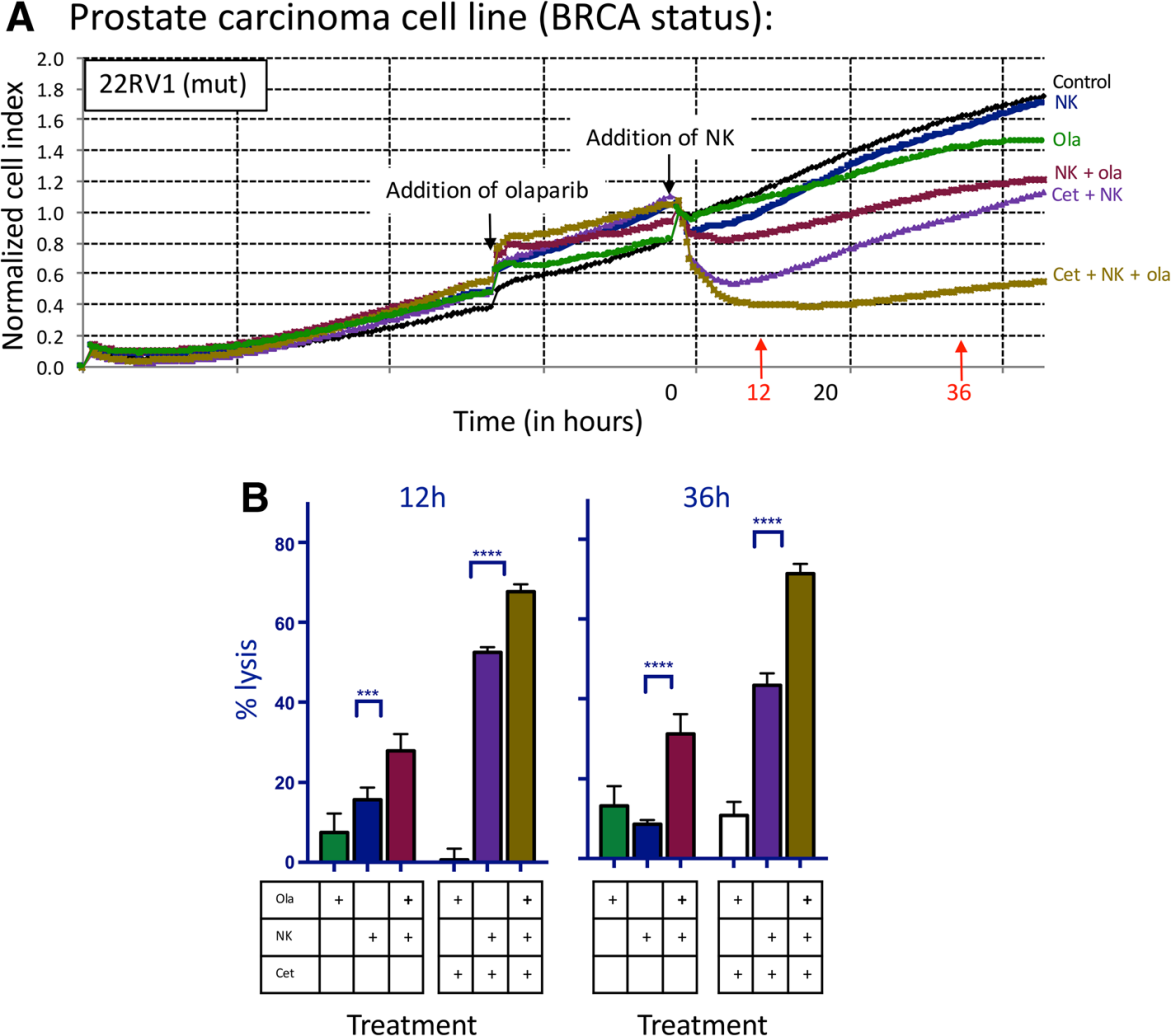
NK-mediated lysis of BRCA mutant prostate carcinoma cells treated with olaparib is further increased by the addition of an ADCC-mediating antibody. **a** Real-time impedance-based cell analysis of BRCA mutant (mut) prostate carcinoma (22RV1) cells in the presence or absence of olaparib (ola) and human NK cells, treated with either cetuximab (cet) or isotype control. **b** 22RV1 cells lysed with or without olaparib pretreatment and NK cells, treated with either cetuximab or isotype control at 12 h and 36 h. This experiment was performed with four different human NK donors with similar results. *p* < 0.001***, *p* < 0.0001****

**Fig. 3:**
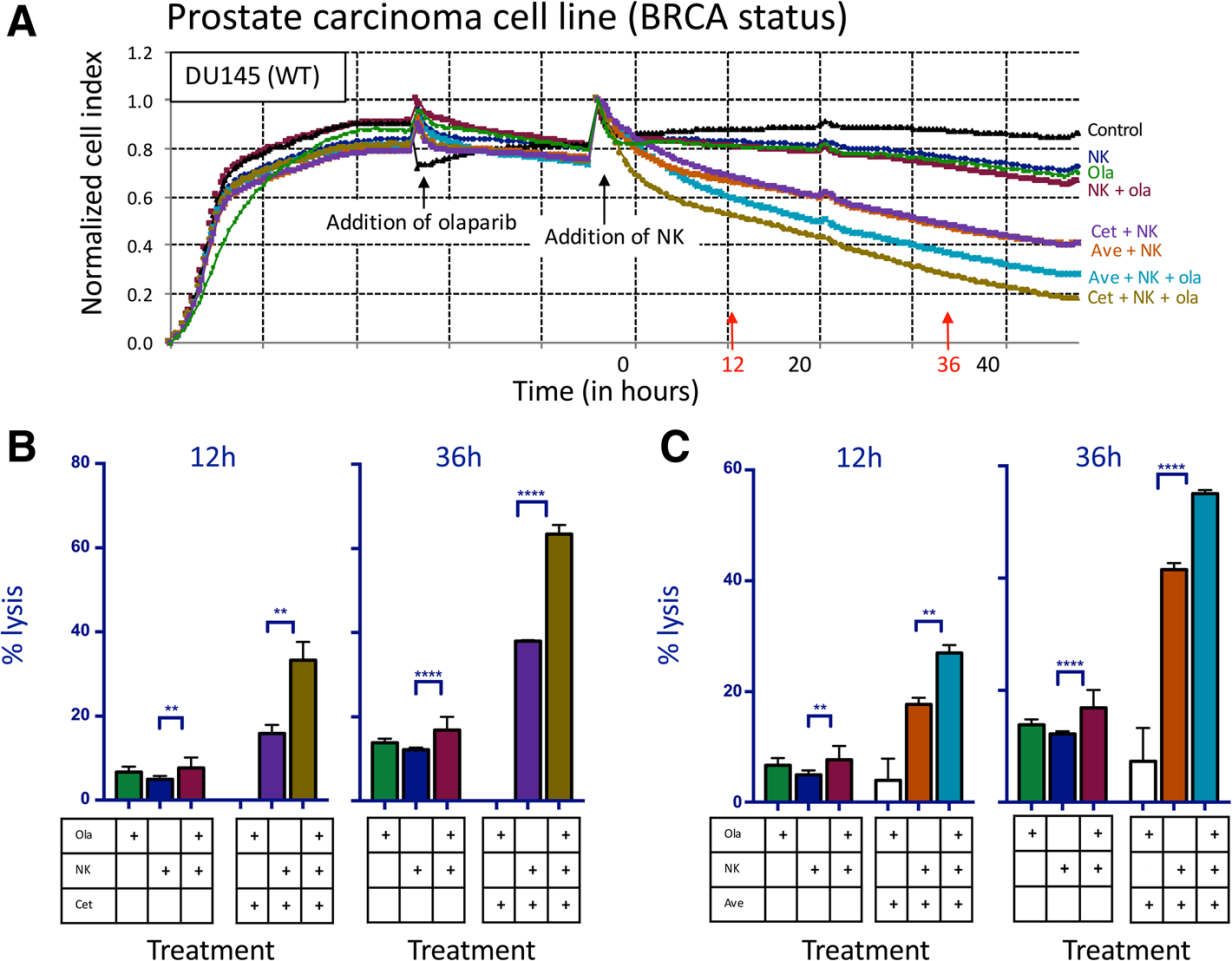
NK-mediated lysis of BRCA wildtype prostate carcinoma cells treated with olaparib is further increased by the addition of ADCC-mediating antibodies. **a** Real-time impedance-based assay of BRCA WT prostate carcinoma (DU145) cells in the presence or absence of olaparib (ola), human NK cells, and mAbs. **b** Lysis of BRCA WT cells with or without olaparib pretreatment and NK cells, and with cetuximab (cet) or isotype control at 12 h and 36 h. **c** Lysis of DU145 cells with or without 24 h pretreatment with olaparib and NK cells, with either avelumab (ave) or isotype control at 12 h and 36 h. These experiments were performed with four different human NK donors with similar results. *p* < 0.01**, *p* < 0.0001****

We next examined the effect of olaparib exposure on NK-mediated ADCC. The EGFR+ line 22RV1 was treated with cetuximab, and the EGFR+ and PD-L1+ line DU145 was treated with either cetuximab or avelumab (Fig. 2a). In the presence of cetuximab, olaparib exposure significantly increased NK-mediated lysis of 22RV1 compared to olaparib only treated cells as early as 12 h (1.2-fold increase, *p < 0.0001*), reaching a 2.8-fold increase at 36 h (*p < 0.0001*) (Fig. 2b). NK-mediated lysis of cetuximab-treated BRCA WT DU145 cells was significantly increased after olaparib exposure (Fig. 3a) by 2.1-fold at 12 h (*p = 0.0034*) and by 1.7-fold at 36 h (*p < 0.0001*) (Fig. 3b). Olaparib- and avelumab-treated cells demonstrated a 1.5-fold increase in NK-mediated lysis at 12 h (*p = 0.0010*) and a 1.3-fold increase at 36 h (*p < 0.0001*) compared to cells treated with only avelumab (Fig. 3c).

### Increased ADCC following olaparib exposure is dependent on CD16 engagement and not mediated by STING-dependent modulation of mAb targets

**Fig. 4:**
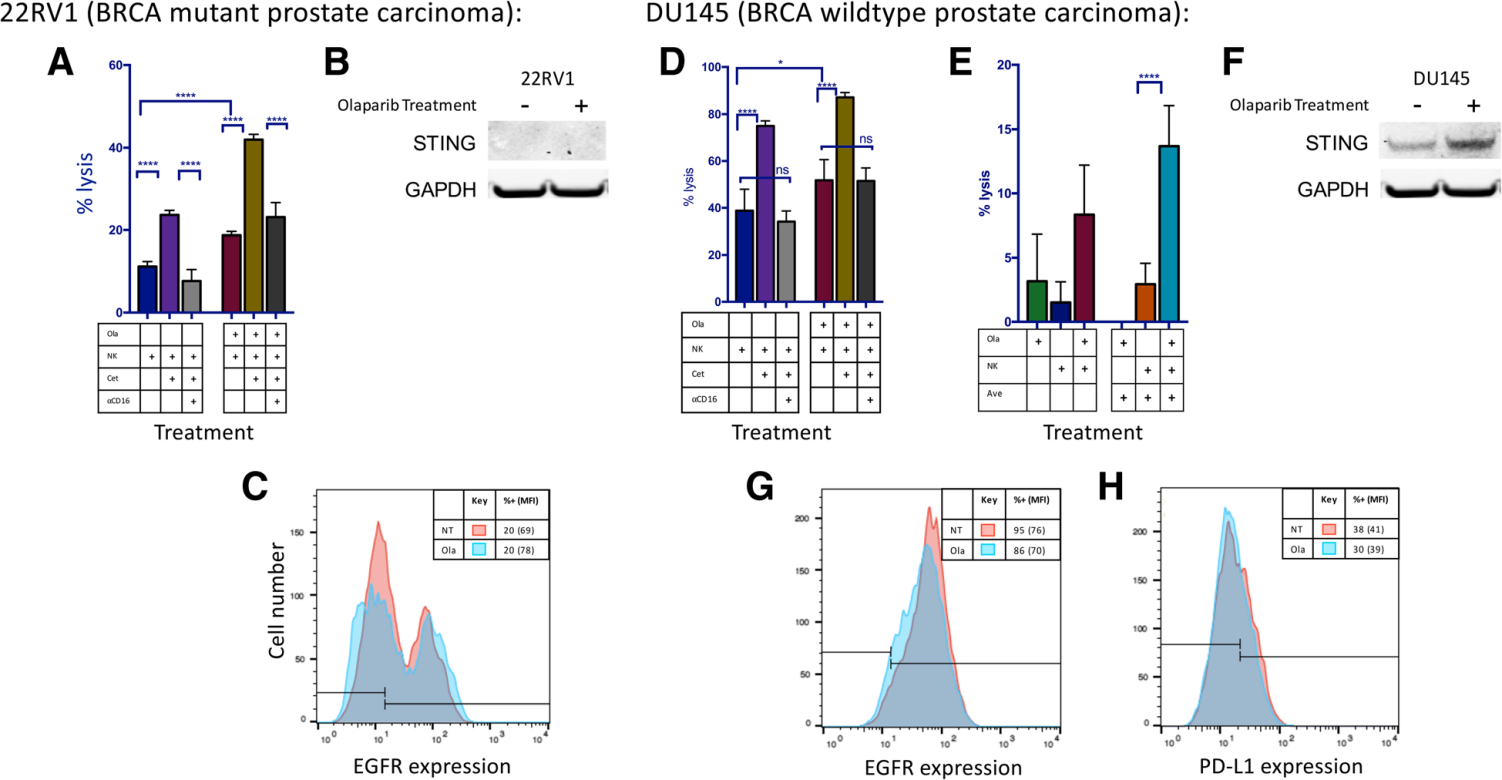
Olaparib treatment enhances ADCC using both cetuximab and avelumab without modulation of mAb targets EGFR and PD-L1. **a** Treatment with cetuximab (cet) significantly increased NK-induced lysis of olaparib (ola)-treated BRCA mutant prostate carcinoma (22RV1) cells at 12 h. The addition of anti-CD16 antibody neutralized this increase, confirming that the increased lysis is attributable to ADCC. **b** STING is not expressed in 22RV1 either before or after olaparib treatment. **c** Olaparib treatment did not result in significant modulation of EGFR expression on 22RV1 cells as measured by flow cytometry. **d** Treatment with cetuximab increased NK-induced lysis of olaparib-treated BRCA WT prostate carcinoma cells (DU145) cells. Role of anti-CD16 antibody on increased lysis attributable to ADCC. **e** The PD-L1+ cell line DU145 also underwent NK-induced ADCC in the presence of the anti-PD-L1 antibody avelumab (ave). Lysis of DU145 cells after 12 h in the presence or absence of olaparib and NK, treated with either avelumab or isotype control is shown. **f** STING was upregulated in DU145 following exposure to olaparib. **g** Olaparib treatment did not result in significant modulation of EGFR expression in DU145 cells as measured by flow cytometry. **h** Olaparib treatment did not result in significant modulation of PD-L1 expression in DU145 cells as measured by flow cytometry. These experiments were performed twice with similar results. *p* < 0.05*, *p* < 0.0001****

To confirm that the increased NK-cell lysis of tumor cells exposed to olaparib in the presence of avelumab or cetuximab is specifically mediated by ADCC, NK effectors were exposed to a CD16-neutralizing antibody prior to being used in an in vitro ADCC assay. Lysis of both olaparib-treated and untreated cells was significantly inhibited in the presence of CD16-neutralizing antibody, confirming that the augmented NK-cell lysis of tumor cells exposed to olaparib in the presence of mAbs is mediated by ADCC in both BRCA mutant (*p < 0.0001*) (Fig. 4a) and BRCA wildtype (*p < 0.0001*) cells (Fig. 4a and d). Functional assays in which NK cells were treated overnight with olaparib and washed before plating were also performed; there was no improved killing of tumor cells, confirming that the PARPi was affecting the phenotype of the target cells rather than the NK cells (Additional file 2: Table S1).

PARP inhibitors have been previously shown to activate the Stimulator of Interferon Genes (STING) pathway, thereby stimulating upregulation of PD-L1 [13]. STING expression was evaluated by Western blot and was not found to be present in BRCA mutant 22RV1 either before or after olaparib treatment (Fig. 4b). STING was present and upregulated, however, after olaparib exposure in BRCA WT DU145 (Fig. 4f).

We interrogated the effect of olaparib treatment on the expression of EGFR and PD-L1, the respective target proteins of cetuximab and avelumab. Target cells were exposed to olaparib prior to analysis for cell-surface expression of these markers. No significant increase in PD-L1 expression was observed in DU145 cells (Fig. 4h). 22RV1 was confirmed to be negative for PD-L1 (baseline expression of 1%, data not shown). Similarly, neither cell line showed a significant upregulation of EGFR (Fig. 4c and g).

### Expression of gene encoding death receptor TRAIL-R2 is upregulated following olaparib exposure

**Fig. 5:**
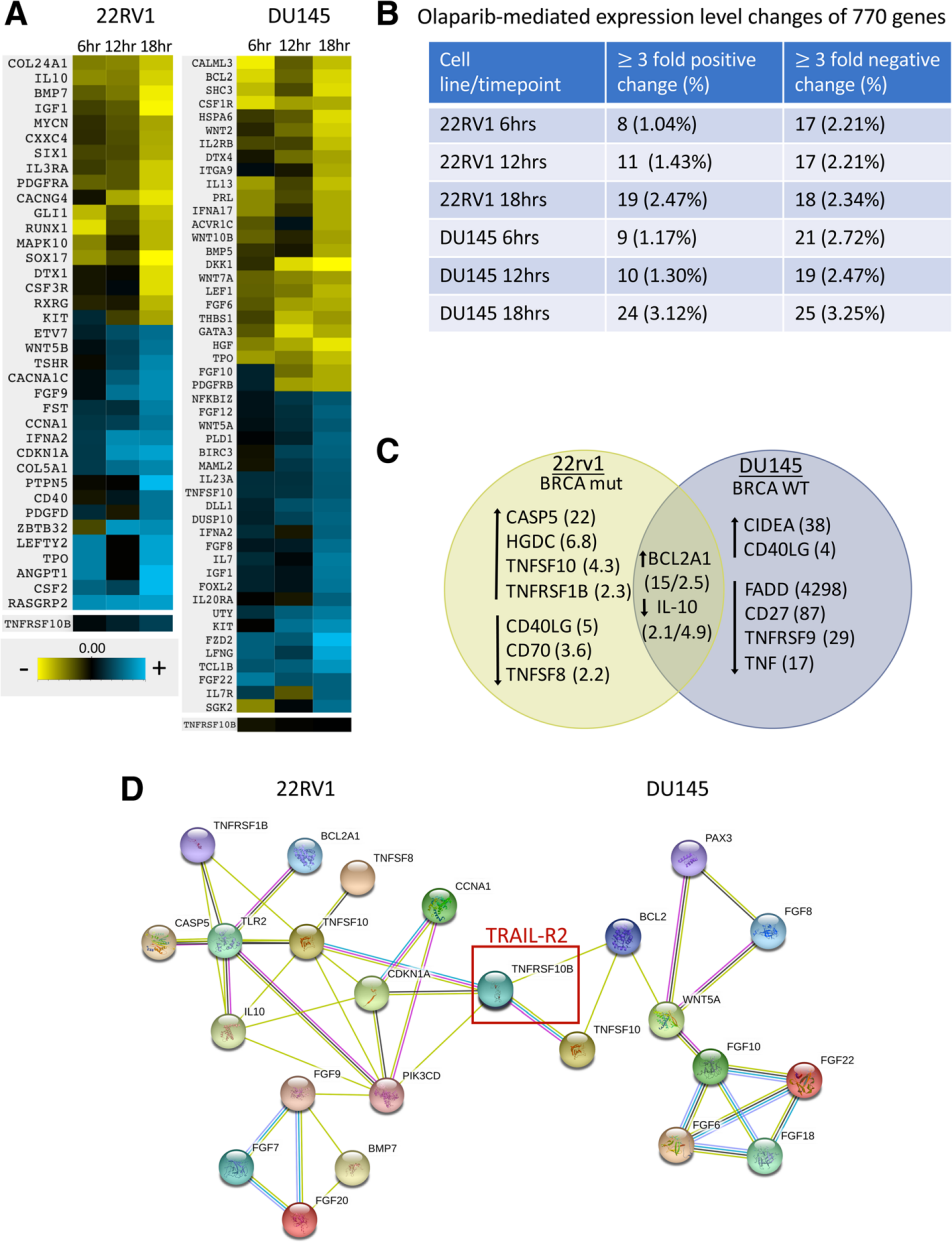
Olaparib treatment induces increased expression of gene TNFRSF10B (TRAIL-R2) in both BRCA mutant and wildtype prostate carcinoma. **a** Heatmap demonstrating genes modulated by at least 3-fold after olaparib treatment as identified by the NanoString nCounter® PanCancer Pathways Panel. TNFRSF10B (TRAIL-R2) was upregulated in both cell lines (by 2-fold in 22RV1 and by 20% in DU145). **b** Summary of modulated genes identified in NanoString analysis. **c** Summary of modulated genes identified in Apoptosis RT^2^ Profiler PCR Array with fold-change denoted in parentheses. **d** STRING network of protein-protein interactions of a curated set of modulated genes identified by both NanoString and apoptotic array. TNFRSF10B (TRAIL-R2; red box) identified as the nexus of both sets

To establish a potential mechanism of increased NK-mediated target cell lysis following olaparib exposure, we performed a NanoString RNA analysis on tumor cells treated with olaparib for 6, 12, and 18 h (Fig. 5a). The initial NanoString screen, utilizing a three-fold cutoff at any timepoint, identified 102 genes of interest in DU145 (67 downregulated and 35 upregulated) and 89 genes of interest in 22RV1 (52 downregulated and 37 upregulated) (Fig. 5b). In addition, an apoptotic array identified genes related to cell growth and apoptosis; 9 select genes (4 downregulated and 5 upregulated) of interest were identified in DU145 and 11 (5 downregulated and 6 upregulated) were identified in 22RV1 (Fig. 5c, Additional file 1: Table S2). Modulation of the tumor necrosis factor superfamily (TNFSF) was observed in both cell lines. Only one downregulated gene was identified, IL-10. IL-10 has a distinct role upregulating human NK function [14]. The apoptotic gene array also identified changes in the expression levels of various members of TNFSF in both cell lines following treatment with olaparib. These data, taken together, indicated that the nexus of these affected genes is death receptor TNFRSF10B (TRAIL-R2), which was found by NanoString to be upregulated by 2-fold in 22RV1 and by 20% in DU145 (Fig. 5d).

### Clinically relevant exposure of prostate carcinoma to olaparib upregulates death receptor TRAIL-R2 and induces caspase cascade

**Fig. 6:**
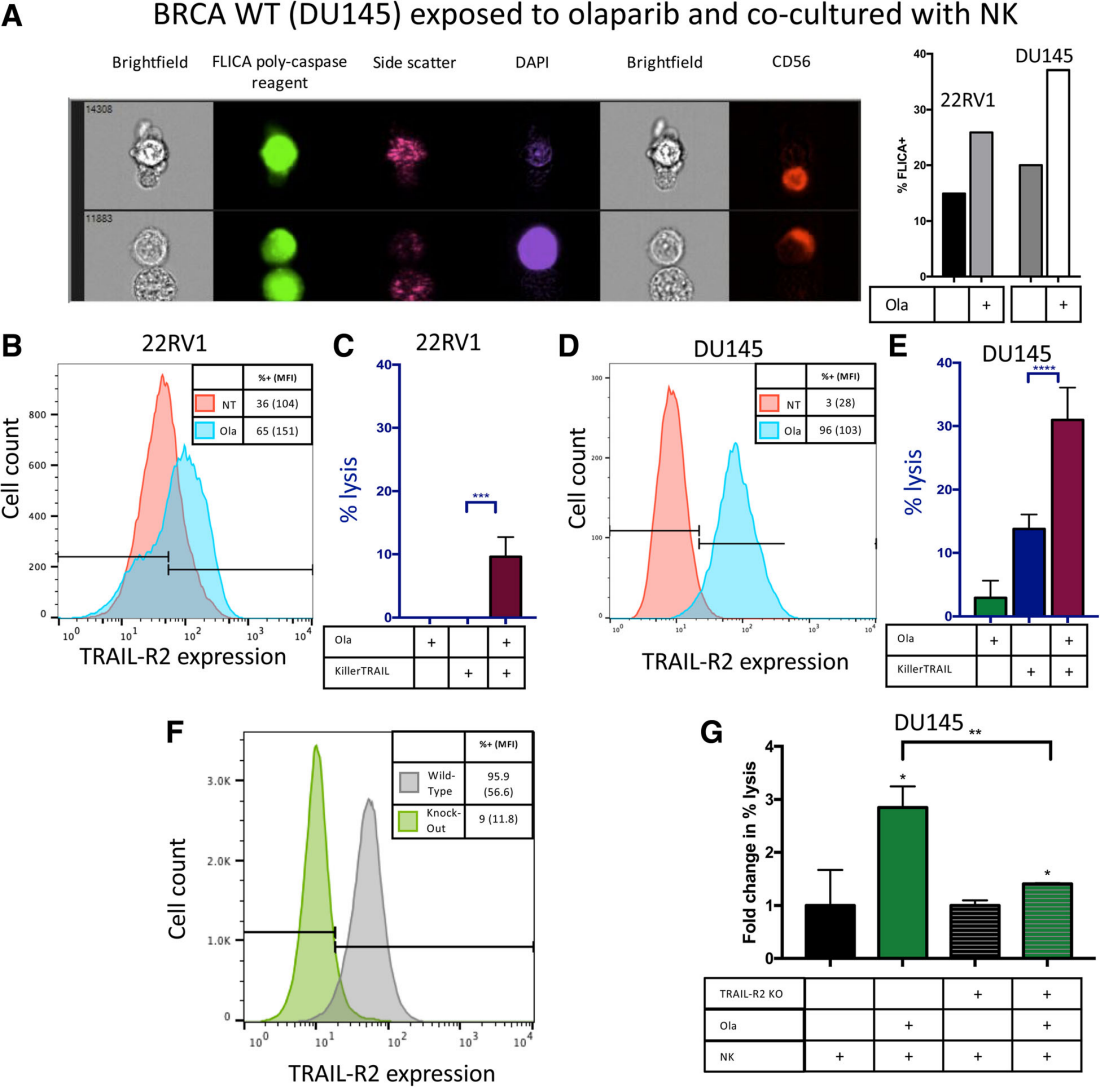
TRAIL-R2 is functionally upregulated upon exposure to olaparib and leads to increased caspase cascade activation. **a** Representative image demonstrated NK cell lysing olaparib-treated BRCA WT target cell by activating caspase cascade and a representative graph demonstrating increased caspase activation as identified by a FLICA reagent following administration of olaparib in both BRCA WT and BRCA mutant cells. **b** Expression of TRAIL-R2 on 22RV1 was upregulated from 36% (MFI 104) to 65% (MFI 151) following a 48 h treatment with olaparib. **c** BRCA mutant prostate carcinoma (22RV1) cell lysis with or without olaparib (ola) and KillerTRAIL antibodies after 36 h. **d** Expression of TRAIL-R2 on DU145 was upregulated from 3% (MFI 28) to 96% (MFI 103). **e** BRCA WT prostate carcinoma (DU145) cell lysis with or without olaparib and KillerTRAIL antibodies after 36 h. **f** Expression of TRAIL-R2 on Wild-Type and TRAIL-R2 CRISPR knockout DU145 cells. **g** Functional confirmation of the involvement of TRAIL-R2. DU145 and TRAIL-R2 CRISPR knockout DU145 cell lysis with or without olaparib and NK cells after 36 h. Depicted is fold-change in percent lysis. These experiments were performed twice with similar results. *p* < 0.05*, *p* < 0.01**, *p* < 0.001***, *p* < 0.0001***

Death receptors activate a caspase cascade that ultimately results in apoptosis [15]. Using imaging flow cytometry, we interrogated the activation of caspases-1, − 3, − 4, − 5, − 6, − 7, − 8 and − 9 in target cells following treatment with olaparib and exposure to human NK cells from healthy donors. Olaparib-treated BRCA mutant 22RV1 cells demonstrated a 1.7-fold increase in caspase activation compared to untreated cells after exposure to two different healthy NK donors. Olaparib treatment increased caspase activation 1.9-fold in BRCA WT DU145 after exposure to the first donor (Fig. 6a), and 1.3-fold after exposure to NK cells from the second donor as shown by fluorochrome inhibitor of caspases (FLICA) reagent positivity. Representative images demonstrate an NK cell lysing a target cell by initiating a caspase cascade (Fig. 6a).

NanoString data indicated RNA-level changes of the expression of TRAIL-R2 on prostate carcinoma cells, and thus we sought to examine the modulatory effect of olaparib on cell-surface expression of TRAIL-R2 using flow cytometry. BRCA mutant cells demonstrated a significant upregulation of TRAIL-R2 following olaparib exposure (*p < 0.001)* (Fig. 6b). We examined whether the upregulation of TRAIL-R2 resulted in functional engagement of the TRAIL pathway in prostate carcinoma cells. Cells treated with or without olaparib were exposed to KillerTRAIL ligand, a recombinant antibody that binds to the TRAIL-R family and self-trimerizes, inducing apoptosis. In BRCA mutant 22RV1, olaparib exposure significantly enhanced KillerTRAIL-induced apoptosis after 36 h (*p = 0.0009*) (Fig. 6c). In BRCA WT cells, olaparib exposure mediated a remarkable 32-fold upregulation of TRAIL-R2 (*p < 0.001*) (Fig. 6d). For BRCA WT DU145, olaparib exposure also significantly enhanced KillerTRAIL-induced apoptosis, with lysis increasing 2.2-fold at 36 h *(p < 0.0001*) (Fig. 6e). To confirm the role of TRAIL-R2 in olaparib mediated enhancement of NK sensitivity, the TRAIL-R2 gene was deleted by CRISPR. The deletion of TRAIL-R2 was confirmed by sequencing and flow cytometry (Fig. 6f). When DU145 wild-type cells were exposed to olaprib, there was a 2.9 fold increase in NK mediated lysis (Fig. 6g) *(p < 0.001*). When TRAIL-R2 CRISPR knockout DU145 cells were exposed to olaprib, there was only a 1.4 fold increase in NK mediated lysis (*p < 0.01*), significantly less than that seen with DU145 wild-type cells *(p < 0.0001*), (Fig. 6g, Additional file 3: Figure S1). These data, taken together, indicate that olaparib exposure mediated a significant increase in surface TRAIL-R2, and that this increase was largely responsible for subsequent increased NK mediated killing.

### Lysis by haNK or NK cells treated with an IL-15/IL-15Rα superagonist can be further enhanced with the addition of olaparib

**Fig. 7:**
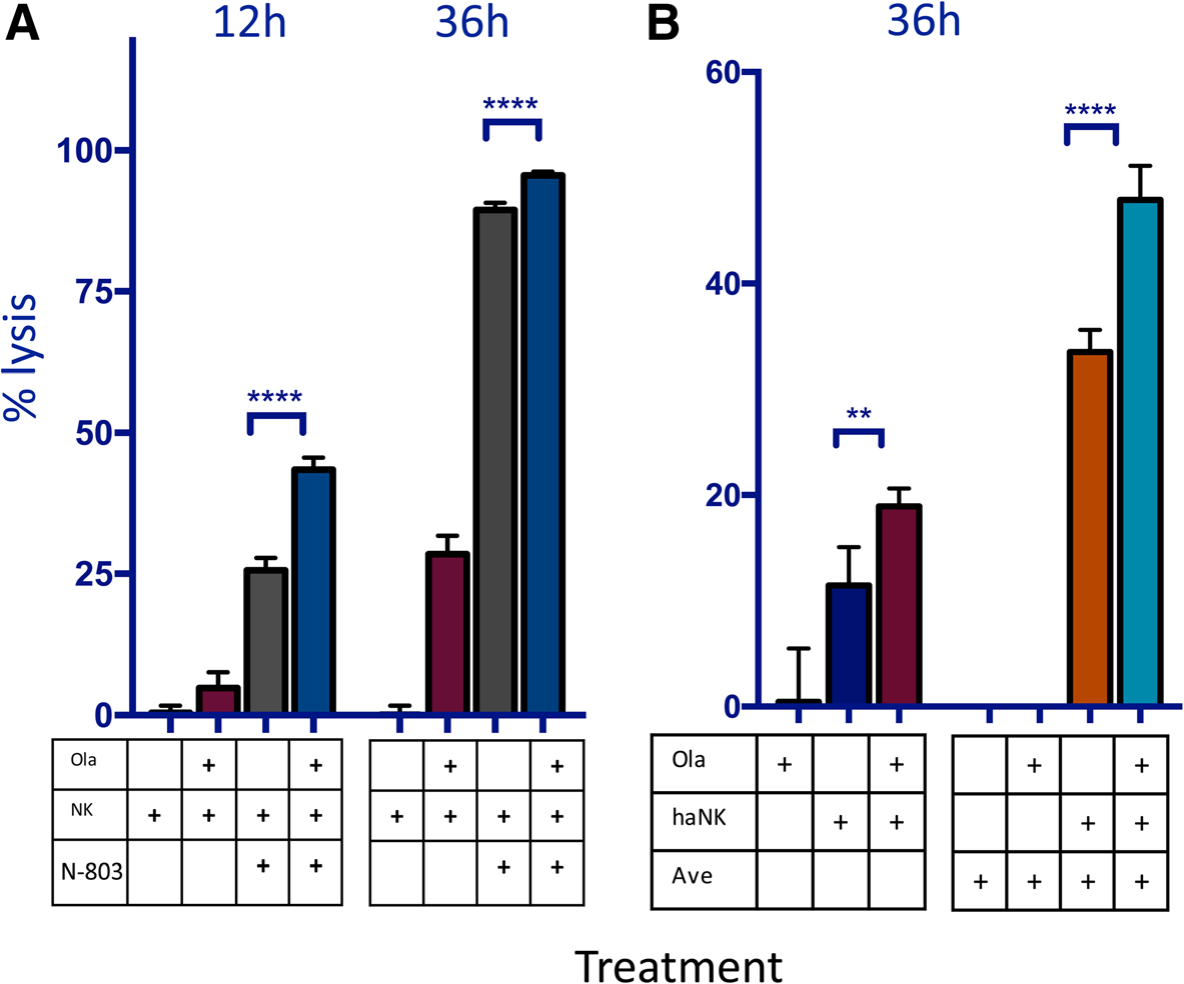
Lysis of target cells by natural killer (NK) cells treated with N-803 or high-affinity NK cells (haNK) can be further increased with the addition of olaparib. **a** Lysis of BRCA wildtype prostate carcinoma (DU145) cells at 12 h and 36 h with or without pretreatment with olaparib (ola) and NK cells incubated in the presence or absence of an IL-15/IL-15Rα superagonist (N-803). **b** Lysis of DU145 at 36 h by haNK cells in the presence or absence of olaparib and avelumab (ave). p < 0.05*, p < 0.01**, p < 0.0001****

We broadened our studies to include two other immunomodulatory agents, haNK cells and N-803 (formerly known as ALT-803). N-803 is an IL-15/IL-15Rα superagonist known to increase the killing capacity of human NK cells. Lysis mediated by N-803-treated NK cells was increased by 1.7-fold at 12 h (*p = 0.0001*) and by pretreating target cells with olaparib; a significant difference persisted at 36 h (Fig. 7a). DU145 cells were also exposed to high-affinity NK cells (haNK) in the presence and absence of olaparib. After 36 h, olaparib-treated cells had 1.7-fold increased lysis (*p = 0.001*) (Fig. 7b). When cells were also exposed to avelumab, lysis was increased 1.4-fold after 36 h (*p < 0.0001*) (Fig. 7b).

### Olaparib increases NK-mediated lysis in an additional diverse set of tumor types

**Fig. 8:**
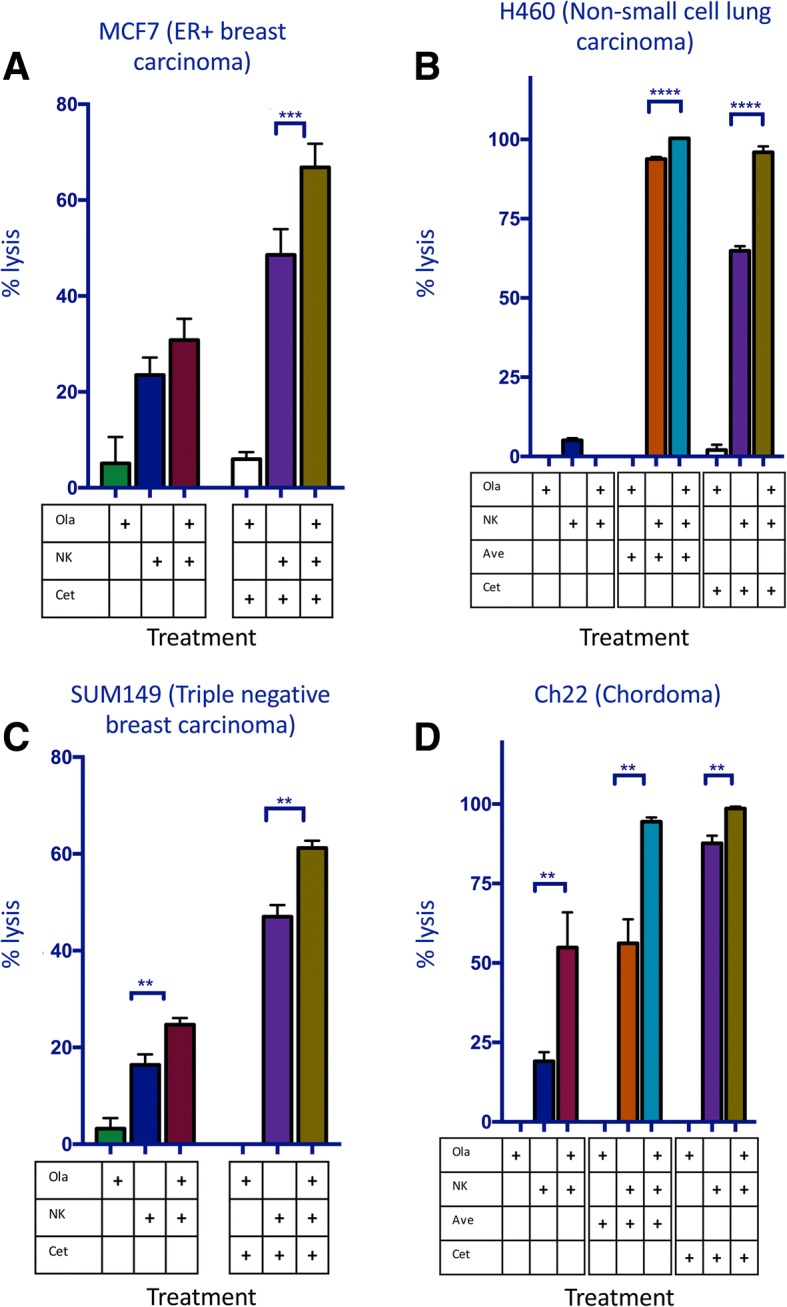
Olaparib increases NK-mediated target cell lysis and ADCC in a diverse set of tumors. A variety of tumor types demonstrated increased NK cell-mediated lysis as measured by real-time impedance. **a** Lysis of estrogen receptor positive (ER+) breast carcinoma (MCF7) in the presence or absence of olaparib and NK, treated with cetuximab or isotype control. **b** Lysis of non-small cell lung carcinoma (NSCLC) (H460) in the presence or absence of olaparib and NK, treated with cetuximab, avelumab (ave) or isotype control. **c** Lysis of triple negative breast carcinoma (SUM149) in the presence or absence of olaparib and NK, treated with cetuximab (cet) or isotype control. **d** Lysis of chordoma (Ch22) in the presence or absence of olaparib and NK, treated with cetuximab, avelumab or isotype control. *p* < 0.01**, *p* < 0.001***, *p* < 0.0001****

The above studies indicate that olaparib may have an immunomodulatory role in prostate cancer as well as its approved indications of ovarian and breast cancer. We further expanded our studies to include additional human cancer cell lines. These included MCF7 (an ER+ breast cancer) and H460 (a non-small cell lung cancer), both of which showed increased NK-mediated lysis of olaparib-treated cells and further increased lysis with the addition of the ADCC-mediating antibody cetuximab (Fig. 8a and b). H460 expresses PD-L1 in addition to EGFR and therefore showed increased ADCC with both avelumab and cetuximab (Fig. 8b). We also investigated two cell lines for which there is no effective standard of care: SUM149 (triple negative breast cancer) and Ch22 (chordoma, a rare sarcoma that originates from the remnants of the notochord). We observed increased NK-mediated lysis of these cells after olaparib treatment (Fig. 8c and d). The EGFR+ line SUM149 showed further increased lysis after the addition of cetuximab (Fig. 8c). Ch22 is both EGFR and PD-L1 positive and demonstrated an increase in lysis after the addition of both cetuximab and avelumab (Fig. 8d).

## Discussion

Recent studies have shown promise for combination therapies utilizing PARP inhibitors and various immuno-oncology modalities together. PARP inhibitors have been previously shown to augment the efficacy of trastuzumab in treating HER2+ breast cancer both in vitro and in vivo [16]. Similarly, cetuximab and PARPi have been shown to enhance DNA damage and cytotoxicity in head and neck cancer cells compared to either treatment alone [17]. However, these studies did not measure immune cell interactions with tumor cells following combination therapy, and thus the synergistic potential of mAbs and PARPi may be even greater than previously thought.

PARP inhibitors are currently approved only for breast and ovarian cancer patients with germline BRCA mutations, but in recent years it has become clear that the applications of PARPi likely also extend to patients with somatic BRCA mutations and other forms of homologous recombination deficiency [18]. A recent phase I/II clinical trial demonstrated that PARPi had similar efficacy in ovarian cancer patients with somatic BRCA 1/2 mutations compared to those with germline mutations [19]. Scott et al. made the clinical observation that the presence of a BRCA mutation is neither necessary nor sufficient for patients to benefit from PARPi therapy, suggesting that PARP inhibitors may have additional mechanisms of action [20]. However, studies examining the usefulness of PARPi as part of combination therapy in homologous-recombination intact cancers are still exploratory. In 2016, Hijaz et al. demonstrated that combination therapy using olaparib and metformin had antitumor activity both in vitro and in vivo in BRCA intact ovarian cancer cells. [21] The following year, a retrospective study of radiological responses to olaparib treatment in patients with a variety of solid tumors demonstrated that among cancer patients with unknown or wildtype BRCA 1/2 status 20.6% had a measurable response to treatment [22]. More recently, olaparib has been investigated specifically in men with BRCA-intact mCRPC. In a recent all-comers trial, olaparib in combination with abiraterone was shown to improve progression-free survival compared to abiraterone alone (8.2 months vs 13.8 months) [23]. Homologous recombination status was available for 56 of the 142 patients, and of those 35 were known to have no homologous recombination deficiency. Although the study was not powered for subgroup analysis, the authors suggested that the addition of olaparib to abiraterone treatment resulted in a similar improvement in progression-free survival of patients regardless of the mutational status of homologous recombination repair genes.

Here, we exposed the BRCA mutant 22RV1 and BRCA WT DU145 prostate carcinoma lines to 20 μM olaparib, a clinically achievable dose based on steady state plasma levels in patients receiving 300 mg BID olaparib [24–26]. We confirmed and expanded upon the observations of Scott et al. [20] by demonstrating that olaparib increased the NK-mediated lysis of prostate carcinoma cells regardless of BRCA phenotype (Figs. 1, 2, 3, 4 and 7). It also enhanced lysis of cells treated with the PD-L1 inhibitor avelumab, which promotes ADCC (Figs. 3, 4 and 7). ADCC occurs when CD16 (FcγRIII) on NK effectors interacts with the Fc portion of IgG1 antibodies that are bound to target cells. It is an important biological phenomenon that may be useful as part of a combination therapy involving other immunomodulatory drugs [27].

Avelumab also has the additional benefit of checkpoint inhibition. Early results of clinical trials using checkpoint inhibitors in mCRPC patients were mixed; however, a phase II trial in 2016 demonstrated reductions in PSA in 3/10 patients and partial responses as measured by RECIST in 2/10 patients after adding pembrolizumab to standard dose enzalutamide, suggesting that PD-L1 blockade should be re-examined in prostate cancer patients [28, 29]. An ongoing phase I/II combination therapy trial utilizing the PD-L1 inhibitor durvalumab plus olaparib has demonstrated favorable PSA responses in 8/17 mCRPC patients (NCT02484404) [2]. Notably, avelumab is the only FDA-approved checkpoint inhibitor that promotes ADCC [30] and thus may have different antitumor activity compared to other checkpoint inhibitors.

Jiao et al. previously demonstrated that PARPi activates the STING pathway, resulting in upregulation of PD-L1 in breast carcinoma [13]. In BRCA WT prostate carcinoma, we observed an upregulation of STING following PARPi exposure but did not find an associated increase in expression of PD-L1 (Fig. 4). This disparity could be due to differences in olaparib exposure; Jiao et al. exposed tumor cells to 10 μm olaparib for 24 h [13], while we utilized 20 μm olaprib for 60 h, a clinically achievable dose based on steady state plasma levels in patients receiving 300 mg BID olaparib [24–26]. In addition, this difference may be attributable to the fact that our study focused on prostate carcinoma instead of breast carcinoma. This may also be beneficial because an upregulation of PD-L1 allows tumors to evade the host immune system by reducing the proliferation of cytotoxic T cells and inhibiting apoptosis in regulatory T cells. Furthermore, the lack of upregulation of PD-L1 in our studies suggests that ADCC-mediating antibodies that do not target PD-L1 (such as cetuximab) may also be exploited in combination with PARPi, giving researchers a variety of combination therapy options to pursue.

NK-mediated immune surveillance and elimination of non-self cells are dictated in part by cell surface death receptors that activate a caspase cascade in the target cell, resulting in apoptosis. One such death receptor is TRAIL-R2, which is targeted by the TRAIL ligand found on NK cells [31]. Our study demonstrated definitive upregulation of TRAIL-R2 at the RNA, protein, and functional levels, as well as increased activation of the caspase cascade in olaparib-treated target cells. Finally, the functional phenotypic and functional consequences of olaparib mediated TRAIL-R2 modulation was confirmed by CRISPR knockout of the TRAIL-R2 gene (Fig. 6).

Clinically, the significant improvement of NK killing of olaparib treated tumor cells can be applied to patients’ endogenous NK cells; but also to patients receiving treatment with IL-15 (N-803) or NK cells for adoptive transfer (haNK cells) as well (Fig. 7). Human NK cells express any combination of two CD16 alleles: the high affinity (ha) valine (V) allele or the lower affinity phenylalanine (F) allele [5]. Clinical studies have demonstrated that patients who are homozygous for the valine allele (*V*/V) have improved survival following administration of ADCC-mediating mAbs compared to patients with the V/F or F/F genotype [32–35]. HaNK cells have been engineered to endogenously express IL-2 and the high affinity valine allele and thus can be used to functionally convert patients to the high-affinity phenotype [5]. They have been previously been shown to increase ADCC of chordoma cells in combination with cetuximab [7] and avelumab [9] when compared to human NK cells expressing the low affinity allele. Furthermore, our studies demonstrated an olaparib-induced increase in NK-mediated lysis of target cells and ADCC in chordoma cells, as well as non-small cell lung carcinoma, triple negative breast carcinoma, and ER+ breast carcinoma (Fig. 8). An exogenous infusion of NK cells like haNK could be correctly timed to take full advantage of the increased immune recognition induced by PARPi.

## Conclusions

Taken together, these studies support combination therapy using ADCC-mediating mAbs and olaparib regardless of BRCA status in a variety of tumor types, either by exploiting patients’ endogenous NK cells or by administering haNK cells to enhance ADCC.

## Additional files

Additional file 1: **Table S2.** Olaparid treated prostate cancer cells: Apoptotic Array Data. (TIF 4709 kb)

Additional file 2: **Table S1.** Target and NK cells treated with or without olaparib were analyzed for expression of cell surface markers by flow cytometry. (TIF 2079 kb)

Additional file 3: **Figure S1.** Knock-out of TRAIL-R2 abrogates the olaparib-mediated increase in NK cell killing of DU145 prostate carcinoma cells. (TIF 2234 kb)

## Abbreviations

ADCC: Antibody-dependent cellular cytotoxicity
APC: Allophycocyanin
Ave: Avelumab
BID: *bis in die*, twice per day
BRCA: Breast cancer susceptibility gene
Cet: Cetuximab
cGAS: Cyclic GMP-AMP synthase
CRADA: Cooperative Research and Development Agreement
CRISPR: Cas9 enzymes together with CRISPR sequences technology used to edit genes
DAPI: 4′,6-diamidino-2-phenylindole
DMEM: Dulbecco modified eagle medium
DNAM1: DNAX accessory molecule 1
EGFR: Epithelial growth factor receptor
EMEM: Eagle’s minimum essential medium
F: Phenylalanine
FACS: Fluorescence-activated cell sorting
Fc: Fragment, crystallizable
FDA: Food and Drug Administration
FLICA: Fluorochrome inhibitor of caspases
haNK: High affinity natural killer
HER2: Human epidermal growth factor receptor 2
IgG: Immunoglobulin G
IL-15Rα: Interleukin 15 receptor,α subunit
IRF-3: Interferon regulator transcription factor 3
mAb: Monoclonal antibody
mCRPC: Metastatic castration resistant prostate cancer
MFI: Mean fluorescence intensity
Mut: Mutant
N-803: IL-15/15-15RA-Fc; ALT-803
NCI: National Cancer Institute
NIH: National Institutes of Health
NK: Natural killer
NKG2D: Natural killer group 2D
Ola: Olaparib
PARP: Poly (ADP-ribose) polymerase
PARPi: Poly (ADP-ribose) polymerase inhibition
PBMC: Peripheral blood mononuclear cells
PCR: Polymerase chain reaction
PD-L1: Programmed death-ligand 1
PE: Phycoerythrin
PSA: Prostate-specific antigen
RECIST: Response Evaluation Criteria in Solid Tumors
RPMI: Roswell Park Memorial Institute medium
RTCA: Real-time cell analysis
STING: Stimulator of interferon gamma genes
STRING: Search Tool for the Retrieval of Interacting Genes/Proteins
TBK1: TANK binding kinase 1
TNF: Tumor necrosis factor
TNFRSF10B: TNF receptor superfamily member 10b
TRAIL-R2: TNF-related apoptosis-inducing ligand receptor
V: Valine
WT: Wild type
