# Correction to: Immunotherapy Utilizing the Combination of Natural Killer– and Antibody Dependent Cellular Cytotoxicity (ADCC)–Mediating Agents with Poly (ADP-ribose) polymerase (PARP) Inhibition

**DOI:** 10.1186/s40425-019-0715-9

**Published:** 2019-09-02

**Authors:** Kathleen E. Fenerty, Michelle Padget, Benjamin Wolfson, Sofia R. Gameiro, Zhen Su, John H. Lee, Shahrooz Rabizadeh, Patrick Soon-Shiong, James W. Hodge

**Affiliations:** 1 Laboratory of Tumor Immunology and Biology, Center for Cancer Research, National Cancer Institute, National Institutes of Health, 10 Center Drive, Room 8B09, Bethesda, MD 20892 USA; 2 EMD Serono, Billerica, MA USA; 3 NantOmics, City, Culver, CA USA

**Correction to: J ImmunoTher Cancer**

**https://doi.org/10.1186/s40425-018-0445-4**

Following publication of the original article [1], an error was noted in the GAPDH in the western blot depicted in Figure 4b. The GAPDH lanes for the experiment have been updated. The corrected Fig. 4 can be seen below.

**Fig. 4:**
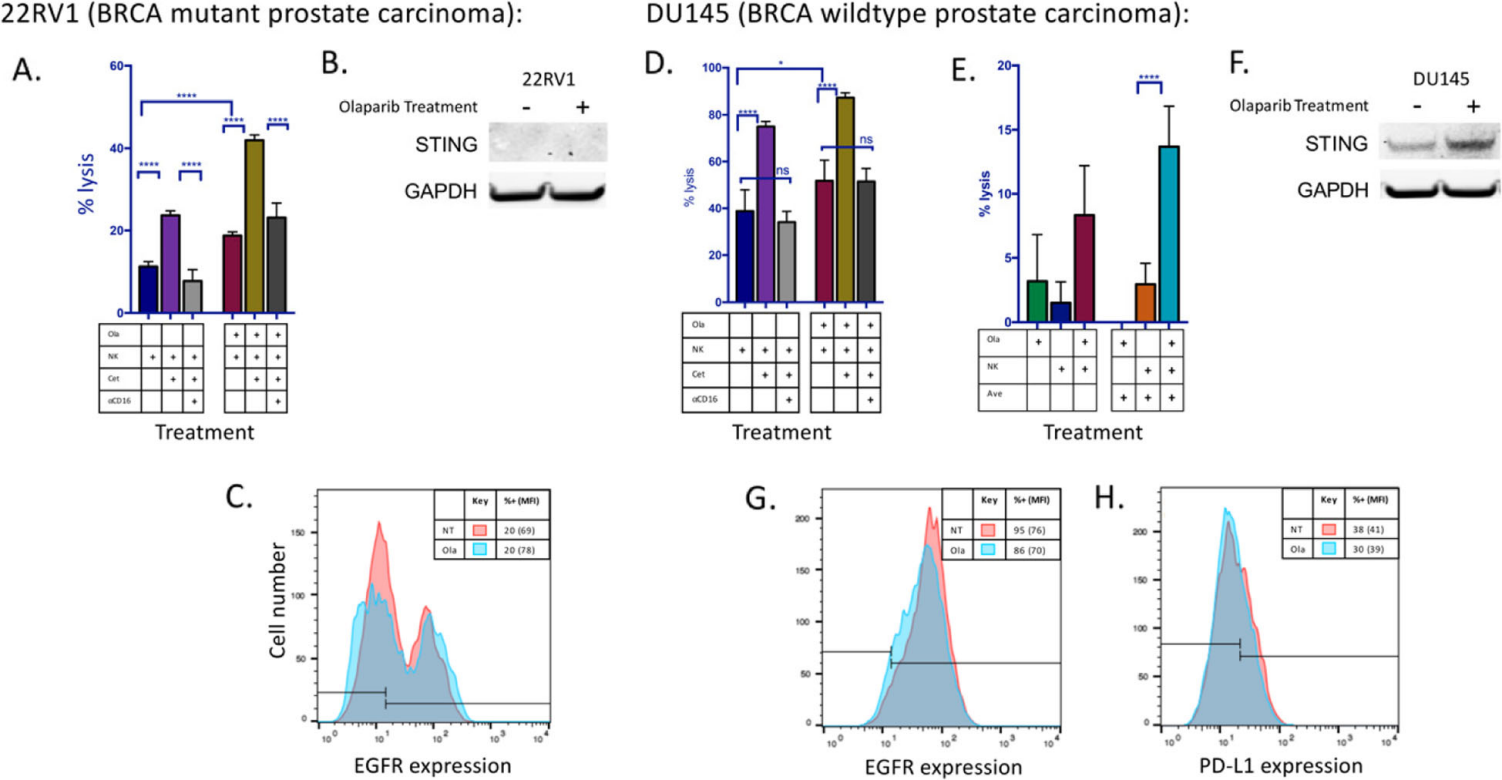
Olaparib treatment enhances ADCC using both cetuximab and avelumab without modulation of mAb targets EGFR and PD-L1. **a** Treatment with cetuximab (cet) significantly increased NK-induced lysis of olaparib (ola)-treated BRCA mutant prostate carcinoma (22RV1) cells at 12 h. The addition of anti-CD16 antibody neutralized this increase, confirming that the increased lysis is attributable to ADCC. **b** STING is not expressed in 22RV1 either before or after olaparib treatment. **c** Olaparib treatment did not result in significant modulation of EGFR expression on 22RV1 cells as measured by flow cytometry. **d** Treatment with cetuximab increased NK-induced lysis of olaparib-treated BRCA WT prostate carcinoma cells (DU145) cells. Role of anti-CD16 antibody on increased lysis attributable to ADCC. **e** The PD-L1+ cell line DU145 also underwent NK-induced ADCC in the presence of the anti-PD-L1 antibody avelumab (ave). Lysis of DU145 cells after 12 h in the presence or absence of olaparib and NK, treated with either avelumab or isotype control is shown. **f** STING was upregulated in DU145 following exposure to olaparib. **g** Olaparib treatment did not result in significant modulation of EGFR expression in DU145 cells as measured by flow cytometry. **h** Olaparib treatment did not result in significant modulation of PD-L1 expression in DU145 cells as measured by flow cytometry. These experiments were performed twice with similar results. *p* < 0.05*, *p* < 0.0001****

The error does not affect the findings of the experiment.
